# Antihyperalgesic Effect of Hesperidin Improves with Diosmin in Experimental Neuropathic Pain

**DOI:** 10.1155/2016/8263463

**Published:** 2016-09-08

**Authors:** Azucena I. Carballo-Villalobos, María-Eva González-Trujano, Francisco Pellicer, Francisco J. López-Muñoz

**Affiliations:** ^1^Laboratorio No. 7 “Dolor y Analgesia” del Departamento de Farmacobiología, Cinvestav-Sede Sur, Calz. de los Tenorios No. 235, Col. Granjas Coapa, CP 14330, México, DF, Mexico; ^2^Laboratorio de Neurofarmacología de Productos Naturales de la Dirección de Investigaciones en Neurociencias, Instituto Nacional de Psiquiatría “Ramón de la Fuente Muñiz”, Av. México-Xochimilco No. 101, Col. San Lorenzo Huipulco 14370, México, DF, Mexico

## Abstract

Neuropathic pain is caused by a primary lesion, dysfunction, or transitory perturbation in the peripheral or central nervous system. In this study, we investigated the hesperidin antihyperalgesic effects alone or combined with diosmin in a model of neuropathic pain to corroborate a possible synergistic antinociceptive activity. Mechanical and thermal hyperalgesia were assessed in the aesthesiometer and plantar tests, respectively, after chronic constriction injury (CCI) model in rats receiving hesperidin (HS, 5 doses from 10 to 1000 mg/kg) alone or combined with diosmin (DS, 10 and 100 mg/kg) in comparison to gabapentin (31.6 mg/kg). UHPLC-MS analysis of cerebral samples was used to recognize the central concentrations of these flavonoids. Participation of different receptors was also investigated in the presence of haloperidol, bicuculline, and naloxone antagonists. Acute hesperidin administration significantly decreased mechanical and thermal hyperalgesia in CCI rats. Antihyperalgesic response of hesperidin, improved by a combination with diosmin (DS10/HS100) in both stimuli, was blockaded by haloperidol, bicuculline, and naloxone, but not WAY100635, antagonists. Both flavonoids were detected in brain samples. In conclusion, hesperidin alone and combined with diosmin produces antihyperalgesic response in the CCI model in rats. Antihyperalgesic effect of DS10/HS100 combination involves central activity partially modulated by D_2_, GABA_A_, and opioids, but not by 5-HT_1A_, receptors.

## 1. Introduction

Neuropathic pain is one of the chronic painful and debilitating conditions which affects large populations worldwide (7% to 18%), with its concomitant disturbance to daily activities [1]. It is defined as “pain initiated or caused by a primary lesion or dysfunction or transitory perturbation in the peripheral or central nervous system” [2]. After nerve injury in preclinical studies, deleterious changes occur in injured neurons and along nociceptive and descending modulatory pathways in the central nervous system, where loss of inhibitory control provokes tactile allodynia and hyperalgesia involving structural changes that increase transmission from A*β* fibers that normally transmit nonpainful stimuli to nociceptive specific second-order neurons in the dorsal horn [3, 4].

Natural products such as flavonoids like hesperidin and diosmin alone or combined have been reported to possess significant anti-inflammatory activity [5–7]. In the case of hesperidin, it is an abundant and inexpensive major plant flavonoid derived from* Citrus* species including sweet orange and lemon [8]. It has shown pharmacological properties, including anti-inflammatory, analgesic [9–11], sedative [12], and antioxidant [13] activities. Its central activities have been associated with GABAergic [12], opioidergic [14, 15], and serotonergic receptors [16]. Its efficacy has also been reported in neuropathy from type 2 diabetic rats [17]. Furthermore, it has been reported to be a protector of brain and sciatic nerve tissues against cisplatin-induced oxidative histological and electromyographical side effects in rats [18].

A pharmaceutical combination of diosmin (450 mg) and hesperidin (50 mg) has been used clinically in the treatment of venous insufficiency [7, 19] as a purified micronized flavonoid fraction that contains 90% diosmin and 10% hesperidin [20]. This combination has also been reported to possess antiplatelet [21], antioxidant [22], and anxiolytic and sedative-like activities [12]. Studies have demonstrated that a combination of diosmin 450/hesperidin 50 inhibits the anti-inflammatory response in models of acute pain by inhibition of PGE_2_, PGF_2*α*_, and TXB_2_ synthesis which results in a decrease in limb swelling [5, 23]. Individual hesperidin was reported to reverse neuropathic pain by controlling hyperglycemia and hyperlipidemia in rats [17]. Moreover, this flavonoid protected against the lesion induced by toxicity in the brain and sciatic nerve [18]. In a similar manner, hesperetin (aglycone of hesperidin) has been reported to produce antihyperalgesic effects in neuropathic rats [24]. However, there is lacking scientific evidence describing the potential antinociceptive activity of this flavonoid alone or in combination in the therapy of neuropathic pain; all these evidences together allow us to hypothesize that acute administration of hesperidin and its combination with diosmin are good options for the relief of neuropathic pain, likely mediated through different mechanisms previously involved in the bioactivity of these two flavonoids such as opioids, GABA_A_, D_2_ dopamine, and 5-HT_1A_ serotonin receptors. In order to confirm the possible efficacy of these flavonoids in the neuropathic pain therapy, the combination used for clinical treatment of venous insufficiency and an equivalent (1 : 1) and a contrasting combination were examined in this study.

## 2. Materials and Methods

### 2.1. Animals

Male Wistar rats weighing 100–120 g (4-5 weeks old at the beginning of the study) were used to practice nerve injury and evaluated at least after 15 days for nociceptive evaluation. Animals were housed in a room under standard conditions, on a 12 h light/dark cycle, with food and water available* ad libitum*. All study procedures followed the Ethical Guidelines for Investigations of Experimental Pain in Animals and were carried out according to a protocol approved by the local Animal Ethics Committee (Projects numbers NC09-3280.3 and NC12-3280) and in compliance with national (NOM-062-ZOO-1999) and international regulations on the care and use of laboratory animals (Publication number 85-23, revised 1985). Study tests were performed during the light phase. The number of experimental animals was kept to a minimum; at the end of the study, they were euthanized with a CO_2_ overdose.

### 2.2. Drugs

Bicuculline, diosmin, gabapentin, hesperidin, naloxone, and WAY100635 were purchased from Sigma, St. Louis, MO, USA. Haloperidol (MP Biomedicals, USA), ketamine (Ketamine; Probiomed S.A de C.V, Mexico), and xylazine (Procin Equus; PISA Agropecuaria S.A de C.V, Mexico) were used in this study too. Drugs were freshly prepared in physiological saline solution (0.9% NaCl, SS) and administered intraperitoneally (i.p.) in a volume of 1 mL/kg of body weight in rats; only hesperidin, diosmin, and haloperidol were suspended in a 0.5% tween 80 in SS solution to improve its administration. Control animals received the same volume of the respective vehicle (i.p). Doses are referred to the free base. For each experimental procedure, groups consisted of at least six rats. Methanol, acetonitrile, and phosphoric and acetic acids purchased from J. T. Baker were used for analytical experiments.

### 2.3. Chronic Constriction Injury (CCI) Induction

The CCI model [25] or sham-procedure was induced in rats as follows: the animals were anesthetized by i.p. injection of ketamine (50 mg/kg) and xylazine (20 mg/kg). Before incision, the right thigh was sterilized with iodine solution (Povidone); next, the right common sciatic nerve was exposed at the level of the mid-thigh and proximal to the sciatic nerve trifurcation. About 7 mm of the nerve was freed from adhering tissue, and four ligatures (with black braided silk 3.0) were loosely tied around the sciatic nerve at 1 mm intervals. The incision was sutured and the wound was cleansed with a crystal violet solution. In sham-operated animals, the sciatic nerve was isolated but not ligated. CCI and sham-operated rats were tested simultaneously. All surgical procedures were performed by the same researcher. Surgery was considered as day 0.

Only rats developing experimental neuropathy (rats responding to both nociceptive stimuli) were used in the study (approximately 80%). A preliminary experiment was carried out before the administration of all treatments (basal) to corroborate initiation of the induction of chronic constriction injury (CCI) in the sciatic nerve 15 days after surgery [25].

### 2.4. Experimental Design for Acute Administration of Hesperidin or Diosmin

In order to know the minimal and maximal efficacy of hesperidin in this model, a dose-response exploration was done using logarithmic increases between doses (10, 100, and 1000 mg/kg) and a 0.25 logarithmic increase between 100 and 1000 mg/kg to complete at least five doses. Then, an acute injection of hesperidin (10, 100, 316.2, 562.3, and 1000 mg/kg, i.p.), diosmin (10 and 100 mg/kg, i.p.), gabapentin (31.6 mg/kg, i.p.), or the vehicle (0.5% tween 80 in saline solution) was given to the neuropathic rats. Measurement of nociceptive behavior was threshold as well as latency of paw withdrawal. Given that antinociceptive response of hesperidin has been reported to remain at least under 2 h after [26], both parameters were assessed at 30, 60, 90, and 120 min after acute administration of each dose.

### 2.5. Antinociceptive Evaluation

#### 2.5.1. Mechanical Hyperalgesia

The mechanical hyperalgesia was determined via assessing paw withdrawal threshold to mechanical stimuli using a Dynamic Plantar Aesthesiometer (Ugo Basile, Italy). The rats were placed in individual transparent acrylic boxes with metal grid floor, inside a temperature controlled room (at about 25°C), and they were acclimatized for 30 min before testing.

The stimulus was applied with a metal filament (0.5 mm diameter) on the skin of the mid-plantar area of the right hind paw, with an increasing force ramp (1 g/s) of up to 50 g in 50 s, starting below the threshold of detection and increasing continuously up until the rat removed its paw. Response in grams was obtained as the average of three consecutive tests performed at least waiting 3 min between measures. Data are expressed as withdrawal threshold.

#### 2.5.2. Thermal Hyperalgesia

The thermal hyperalgesia was tested with the Hargreaves assay (Ugo Basile, Italy) [27]. For the study, rats were acclimated individually in Plexiglas chambers with heated glass floor for 30 min. With this method, a radiant heat source with a locator light is positioned under the plantar surface of a rodent. The intensity of the lamp was set at 60 Hz and a cut-off of 30 s was used to avoid tissue damage. The light beam was directed at the plantar surface of the right hind paw until the rat responded or for 30 s, whichever occurred first. Latency to withdrawal was measured with three consecutive thermal tests at least 3 min each one. The means of the three tests were estimated. Latency to paw withdrawal was recorded with a built-in timer, which displayed reaction time in 0.01 s increments. Data are expressed as withdrawal latency.

### 2.6. Tissue Sample Preparation

Rats received acute administration of hesperidin at 10, 100, and 1000 mg/kg, i.p., and after 30 and 120 min of the treatments they were anesthetized with CO_2_ to avoid animal stress. Next, they were decapitated to remove the complete brain in order to corroborate its presence at central level. Tissue was immediately stored at −80°C until being processed.

A 10x phosphate buffer saline (PBS, pH 7.4) was prepared by mixing and dissolving the following substances: Na_2_HPO_4_ (anhydrous) 10.9 g; NaH_2_PO_4_ (anhydrous) 3.2 g; NaCl 90 g in distilled water (1000 mL). This buffer (10x PBS) was stored at room temperature and diluted 1 : 10 to obtain 1x PBS used in the experiments. Tissue samples were homogenized in 1 mL of ice-cold 1x PBS per gram of tissue and centrifuged at 20,000 ×g for 30 min. Rat brain supernatants were analyzed to detect the presence of hesperidin and/or diosmin at supraspinal level by UHPLC-MS analysis.

### 2.7. Ultra-High Performance Liquid Chromatography Coupled Masses (UHPLC-MS) Analysis of Flavonoids Concentrations

#### 2.7.1. Standards

Stock, blank material, and standard solutions of hesperidin were prepared in DMSO-methanol 1 : 1 and filtered through an Acrodisc Syringe filter (13 mm, 0.2 *μ*m, GHP minispike; Waters, USA). Standard curves were prepared by spiking blank milli Q water to yield final concentrations of 0.0019, 0.0076, 0.03, 0.488, 1.95, and 500 *μ*g/mL of diosmin and hesperidin.

#### 2.7.2. Tissue Samples

Supernatants of brain samples from sham or vehicle groups were screened prior to spiking to ensure that they were free from hesperidin. All solutions obtained from samples were stored at −20°C until their chromatographic analysis. Prior to spiking in the UHPLC injector, 100 *μ*L of supernatant brain samples was transferred to 300 *μ*L of 1% formic acid in acetonitrile and filtered with an Ostro sample preparation ANSI-96-well-2 mL plate (Ostro Waters, Ireland) at 11,250 mmHg in vacuum conditions for 60 s. Samples were maintained at 15°C in the injection camera during the analysis.

#### 2.7.3. UHPLC-MS Conditions

Chromatographic analysis was performed using ultra-performance liquid chromatography equipment (UHPLC 594G, Acquity Waters, Singapore). The separation was done by employing an Acquity UHPLC HSS T3 EC-C18 column 2.1 × 100 mm, 1.8 *μ*m (Acquity Waters, Singapore) with a thermostat at 35°C. It was coupled to a single-quadrupole mass spectrometer (Acquity QDa detector; Waters, Milford, MA, USA) fitted with electrospray ionization (ESI) in the negative mode. A mixture of 1% acetic acid in milli Q water (A) and pure methanol UHPLC grade (B) was used as mobile phase. Gradient elution was carried out at a constant flow of 0.5 mL/min. The following gradient was applied: 0 min 98% A (2% B), 0–8 min 27% A (73% B), 8–8.5 min 0% A (100% B), 8.5–10 min 0% A (100% B), 10–10.5 min 98% A (2% B), and 10.5–13 min 98% A (2% B). The return to the starting gradient composition (98% A and 2% B) was performed at 10.5 min. The injected volume was 10 *μ*L. Subsequently, the molecular weights of diosmin and hesperidin were accurately determined with a mass spectrometer (MS) operated with dual ESI source in the negative mode. Data were collected in scan MS mode over the* m/z* range from 260 to 610 Da. ESI conditions were set as follows: acquisition SIR, cone voltage 5 V, probe 600°C, capillary 0.8 kV, and sampling frequency 5 Hz, with nitrogen as nebulizer gas. Data acquisition, data handling, and instrument control were performed by the Empower® 3 software (Waters, Milford, MA, USA).

### 2.8. Diosmin/Hesperidin Combination after Acute and Repetitive Administration

The individual and subeffective doses of hesperidin (10 and 100 mg/kg, i.p.) obtained in the acute administration were selected to explore its antihyperalgesic response in combination with individual doses (10 and 100 mg/kg, i.p.) of diosmin. The evaluated combinations of diosmin (DS) and hesperidin (HS) in mg/kg, i.p. were DS10/HS100, DS100/HS10, and DS100/HS100 in acute administration compared with gabapentin (31.6 mg/kg, i.p.) and vehicle groups. The measurement of nociceptive behavior was the same as used in the individual acute treatment of hesperidin or diosmin.

To explore the antihyperalgesic response of these flavonoids in repeated treatment, the combination showing a supra-additive response in both stimuli (DS10/HS100) was used. Hence, this combination or vehicle was daily intraperitoneally administered to rats (at midday) after antinociceptive assay from the 15th day until 27th day of the CCI surgery, and then nociceptive behavior was assessed on days 15 (basal), 18, 21, 24, and 28 to receive 3, 6, 9, and 13 accumulative administrations of treatment, respectively.

### 2.9. Mechanism of Action in the Synergic Antihyperalgesic Interaction after Acute Administration of Diosmin/Hesperidin

Participation of the opioids, GABA_A_, dopamine, and serotonin receptors in the DS10/HS100 combination-induced antihyperalgesic effects were investigated; all these targets have been already reported in individual pharmacological effects of these two flavonoids [12, 14, 15, 26], and their participation is also known in the descending modulation of pain. In this study, in order to look for a more specific participation of these receptors only the combination producing significant response in both antinociceptive parameters was explored after its acute administration. The antagonists naloxone (a nonselective opioid, 1 mg/kg, i.p.), bicuculline (a selective GABA_A_ antagonist, 5 mg/kg, i.p.), haloperidol (a nonselective antagonist D_2_, 0.1 mg/kg, i.p.), and WAY100635 (a selective 5-HT_1A_ antagonist, 0.12 mg/kg, s.c.) were injected immediately after basal behavior registration. Antagonists were given 10 min before the administration of the DS10/HS100 combination or vehicle. Behavioral evaluations were also carried out at 30 and 120 min after treatment administration.

### 2.10. Sedative-Like Response

To explore the influence of diosmin, hesperidin, or its DS10/HS100 combination on sedative-like activity, the open-field test modified by Hemsley and Hopwood [28] was employed in rats 30 min after treatment administration. This consists of a brightly-lit, rectangular, plastic enclosure divided into 9 zones in a 3 × 3 grid formation, and each zone is 10 cm × 10 cm. Rats were placed in the center and were allowed to explore the field for 3 min. The operator manually recorded the number of explorations of the four paws of rats in each square. The total activity parameter was calculated as the sum of the explorations.

### 2.11. Statistical Analysis

Antihyperalgesic effects are described in the corresponding figures as the withdrawal threshold or withdrawal latency in the acute and repeated treatment of hesperidin alone or in combination with diosmin. Each point in the time course curves represents the average of at least 6 animals, and each measure was registered every 30 min during a 120-minute period (acute treatment) or every three days during a 14-day period (repeated treatment). Dose-response effects were obtained from the time course curves by including all the time points observed during the whole period of 120 min or 14 days, respectively. Bars express the mean ± SEM of five time points. Data are described as theoretical sum for the expected additive-effects and then compared to those obtained from the experimental results. Data were analyzed using one-way ANOVA followed by Dunnett's* post hoc* test for multiple comparisons of groups or Student's* t*-test comparing means of two groups. The two- or three-way ANOVA was used to determine if there is an interaction effect between two or three independent variables on a continuous dependent variable, respectively. *P* values < 0.05 were considered to be statistically different. Statistical analysis was carried out using the GraphPad Prism version 5.0 (GraphPad Software, San Diego, CA, USA) and/or SigmaStat version 4.0 (Systat Software, Inc., Chicago, USA) for Windows.

## 3. Results

### 3.1. Acute Hesperidin Reduces Experimental Pain-Associated Behavior in CCI Rats

Withdrawal thresholds and latencies, 15 days after CCI and before treatments, were similar for all the groups and significantly different in comparison to sham rats (*F*_(7,40)_ = 10.60, *P* < 0.001). Injection of vehicle did not change the mechanical withdrawal threshold or thermal withdrawal latency.

**Figure 1:**
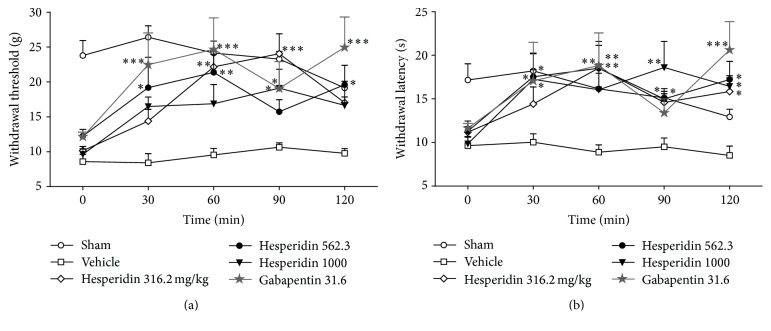
Effects of acute treatment with hesperidin on mechanical and thermal hyperalgesia after inducing CCI in rats. Time course curves show the effect of hesperidin, gabapentin, and vehicle on the withdrawal threshold after mechanical stimulus (a) and on the withdrawal latency after thermal stimulus (b), assessed every 30 minutes over a 2 h period. Each point is the average of withdrawal threshold (g) or withdrawal latency (s) ± SEM in at least six rats. Two-way ANOVA followed by Tukey's test, asterisks indicate statistical significance between the vehicle and the treatment groups on the same day: ^*∗*^ *P* < 0.05, ^*∗∗*^ *P* < 0.01, and ^*∗∗∗*^ *P* < 0.001.

Treatment with hesperidin significantly modified nociceptive parameters associated with dosage (*F*_(7,40)_ = 10.60, *P* < 0.001), time (*F*_(4,40)_ = 10.61, *P* < 0.001), and interaction (*F*_(28,40)_ = 2.07, *P* = 0.003) (Figure 1) as follows: a significant enhancement in the mechanical withdrawal threshold was produced in the presence of hesperidin at 316.2 mg/kg at both 60 and 90 min and 562.3 mg/kg at 30, 60, and 120 min and with the dose of 1000 mg/kg at 90 min. Gabapentin, a dosage of 31.6 mg/kg, showed significant differences at 30, 60, 90, and 120 min (Figure 1(a)). Dosage of hesperidin at 10 and 100 mg/kg did not show significant differences at any time, except by 100 mg/kg at 90 min.

With regard to thermal hyperalgesia, the withdrawal latency was significantly improved after hesperidin treatment with the dosage of 316.2 mg/kg at 60, 90, and 120 min and 562.3 mg/kg at 30 to 120 min and with the dose of 1000 mg/kg at 30, 90, and 120 min. Similar responses were observed for gabapentin administration, which decreased the withdrawal latency at 30, 60, and 120 min (Figure 1(b)). Two-way analysis of variance of the withdrawal latency showed significant main effects of dose (*F*_(7,40)_ = 4.686, *P* < 0.001) and time (*F*_(4,40)_ = 6.710, *P* < 0.001), but there was no significant difference between dose × time interactions (*F*_(28,40)_ = 1.514, *P* = 0.059). In a similar manner, dosage of hesperidin at 10 and 100 mg/kg did not show significant differences at any time.

### 3.2. Cerebral Concentration of Hesperidin

**Figure 2:**
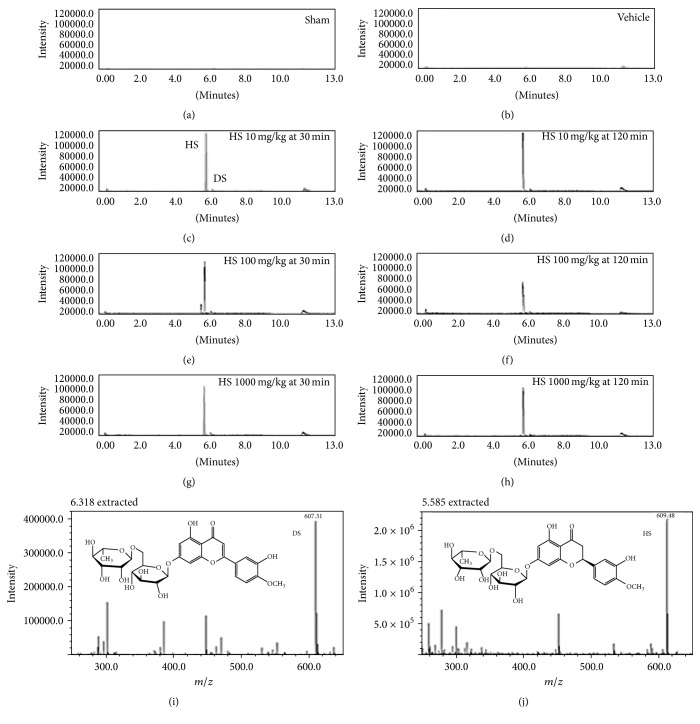
A representative UHPLC-QDA chromatogram quantifying hesperidin in cerebral supernatant of rats. Peaks observed at a retention time of 5.6 min and 6.3 min for hesperidin and diosmin are in accordance with the standards, respectively. Panels (a) and (b) show the chromatograms of control samples from sham and vehicle groups. Chromatograms of hesperidin at acute dosages of 10 mg/kg, i.p. at 30 min (c) and 120 min (d); 100 mg/kg, i.p. at 30 min (e) and 120 min (f); and 1000 mg/kg, i.p. at 30 min (g) and 120 min (h). Mass spectrum describing the molecular weight of diosmin (i) and hesperidin (deprotonated molecular ion [M-H]^−^) (j) and the corresponding retention times.

Chromatographic analyses of homogenates of brain samples treated with hesperidin showed the presence not only of this flavonoid but also of diosmin in comparison to their absence in samples from sham or vehicle groups (Figures 2(a) and 2(b)). Chromatograms obtained for hesperidin at doses of 10, 100, and 1000 mg/kg, i.p. after 30 min (Figures 2(c), 2(e), and 2(g), resp.) and 120 min (Figures 2(d), 2(f), and 2(h), resp.), exhibited two peaks corresponding to diosmin and hesperidin. These compounds were identified by comparing their retention times and UV spectra of the peaks with those of available reference standards and confirmed by spiking the matrix with isolated compounds.

Identified peaks were also analyzed with the deprotonated molecular ion [M-H]^−^ method for diosmin at* m/z* 607 and retention time of 6.3 min (Figure 2(i)) and hesperidin at* m/z* 609 and retention time of 5.6 min (Figure 2(j)). Higher concentrations of hesperidin were detected during the first 30 min at doses of 10 (3.35 *μ*g/mL), 100 (3.36 *μ*g/mL), and 1000 (3.25 *μ*g/mL) mg/kg, i.p. followed by diosmin at dosages of 10 (2.54 *μ*g/mL), 100 (3.24 *μ*g/mL), and 1000 (4.66 *μ*g/mL) mg/kg. In addition, samples from 120 min showed similar concentrations of hesperidin at doses of 10 (4.19 *μ*g/mL), 100 (2.53 *μ*g/mL), and 1000 (3.02 *μ*g/mL) mg/kg, compared to diosmin at doses of 10 (3.78 *μ*g/mL), 100 (7.26 *μ*g/mL), and 1000 (4.94 *μ*g/mL) mg/kg.

### 3.3. Pharmacological Interaction of Acute Administration of Diosmin/Hesperidin on the CCI Model

Given that the analytical assay revealed the presence of diosmin in samples of hesperidin and since these compounds are used as a combination in the clinic, a positive pharmacological interaction between these two flavonoids on neuropathic pain was explored by using the CCI-induced hyperalgesia model.

**Figure 3:**
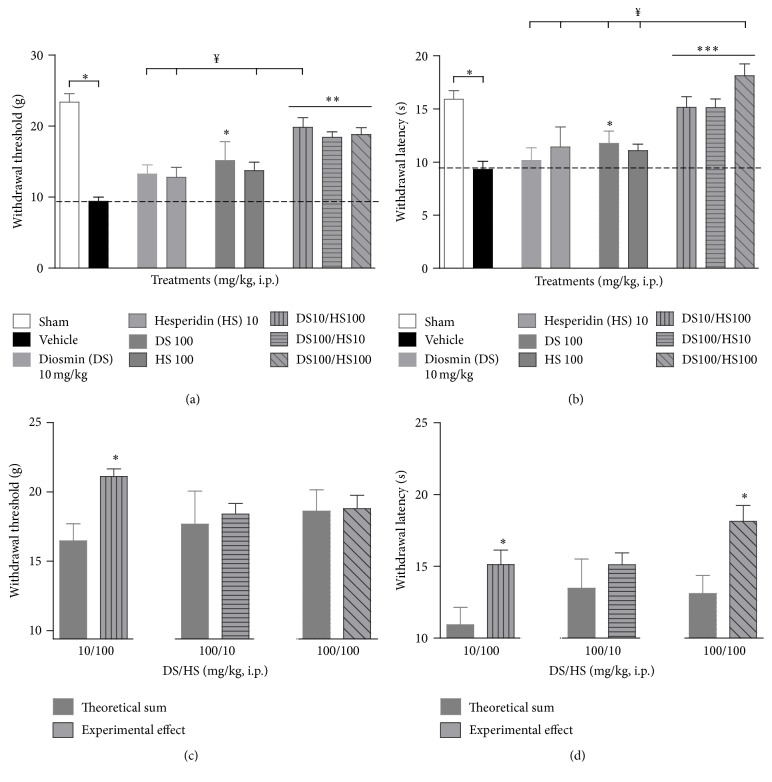
Effects of acute treatment of single hesperidin (HS, 10 and 100 mg/kg) and diosmin (DS, 10 and 100 mg/kg) in comparison with their combinations (DS10/HS100, DS100/HS10, and DS100/HS100 mg/kg) on the mechanical and thermal hyperalgesia after CCI surgery in rats. Column graphs show the effect of hesperidin, diosmin, these flavonoid combinations, and control groups (sham and vehicle) on the withdrawal threshold after mechanical stimulus (a) and on the withdrawal latency after thermal stimulus (b). To analyze the effect of the combinations, the antihyperalgesic response of each one in mechanical (c) and thermal (d) stimuli was contrasted with its respective theoretical antihyperalgesic value (represented from the maximal response obtained in the vehicle group, - - -, (a-b)) and also compared to the effect obtained experimentally (c-d). Each column is the average value of the withdrawal threshold or withdrawal latency ± SEM in at least six rats. For panels (a) and (b), one-way ANOVA followed by Tukey's test, symbols indicate statistical significance, ^*∗∗*^ *P* < 0.01 and ^*∗∗∗*^ *P* < 0.001 versus vehicle, whereas ^*¥*^ *P* < 0.001 for comparison among combinations versus individual treatment. For panels (c) and (d), Student's* t*-test, ^*∗*^ *P* < 0.01.

A combination of these two flavonoids (10 and 100 mg/kg) was analyzed. We used the proportions 1 : 1, 1 : 10, and 10 : 1 denoted as DS100/HS100, DS10/HS100, and DS100/HS10, respectively, in mg/kg, ip. It was observed that the three combinations showed a significant increase in the withdrawal threshold and latency of the mechanical (*F*_(6,35)_ = 4.567, *P* < 0.001) and thermal (*F*_(7,40)_ = 5.535, *P* = 0.0004) stimuli compared to vehicle (9.39 ± 0.60 g and 9.31 ± 0.77 s, resp.) and their individual groups (Figures 3(a) and 3(b)), respectively, whereas, a comparison between the theoretical sums as an additive effect expected displayed a significant supra-additive effect for DS10/HS100 on both mechanical (*t*_(8)_ = 2.438, *P* = 0.0407) and thermal (*t*_(8)_ = 2.673, *P* = 0.0282) stimuli, the combination of DS100/HS100 showed a supra-additive effect (*t*_(8)_ = 2.322, *P* = 0.0427) only in the thermal stimulus, and finally DS100/HS10 demonstrated an additive effect in both stimuli (Figures 3(c) and 3(d)).

**Figure 4:**
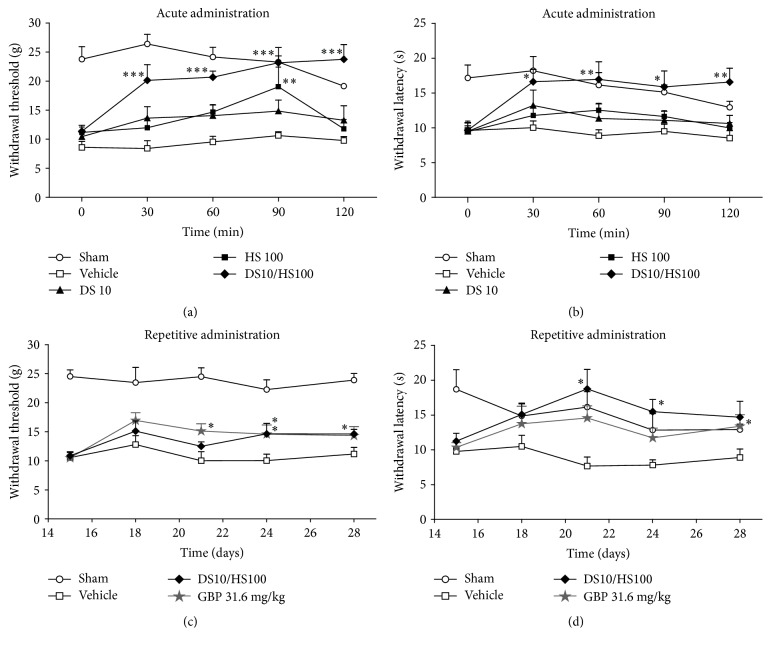
Effect of acute and repetitive treatments of the DS10/HS100 mg/kg dose on mechanical and thermal hyperalgesia after inducing CCI in rats. Time course curves show the effect of an acute dose of diosmin 10 mg/kg and hesperidin 100 mg/kg in comparison with both their combination DS10/HS100 mg/kg and vehicle on the withdrawal threshold after mechanical stimulus (a) and on the withdrawal latency after thermal stimulus, assessed every 30 minutes over a 2 h period (b). Panels (c) and (d) show the effect of daily repetitive administration of a combination of DS10/HS100 mg/kg, gabapentin (GBP), and vehicle on both withdrawal threshold after mechanical stimulus (c) and withdrawal latency after thermal stimulus (d), assessed on days 15, 18, 21, 24, and 28 after surgery. Each point is the average of the withdrawal threshold (g) or withdrawal latency (s) ± SEM in at least six rats. Two-way ANOVA followed by Tukey's test, asterisks indicate statistical significance among the vehicle and the treatment groups on the same day: ^*∗*^ *P* < 0.05, ^*∗∗*^ *P* < 0.01, and ^*∗∗∗*^ *P* < 0.001.

The time course of the antihyperalgesic effect of individual diosmin (10 mg/kg) and hesperidin (100 mg/kg), significant for the second flavonoid at 90 min (Figure 4(a)), in comparison to the combination (DS10/HS100) and vehicle groups confirmed a supra-additive response of these flavonoids by increasing significantly the withdrawal threshold (Figure 4(a)) and withdrawal latency (Figure 4(b)) in the experimental neuropathic rats. This antihyperalgesic response remained during the length of the study (120 minutes), reaching even the basal response of the group of sham rats for every time tested (Figures 4(a) and 4(b)). Two-way ANOVA showed significant effects of the nociceptive mechanical paw withdrawal threshold, on dose (*F*_(4,25)_ = 22.971, *P* < 0.001), treatment time (*F*_(4,25)_ = 8.204, *P* < 0.001), and dose × time interactions (*F*_(16,25)_ = 3.349, *P* < 0.001). In the case of the withdrawal latency, there is a significant effect because of dose (*F*_(4,25)_ = 8.871, *P* < 0.001) and time (*F*_(4,25)_ = 3.837, *P* = 0.006), but not by interaction of dose × time (*F*_(16,25)_ = 1.608, *P* = 0.080).

### 3.4. Effect of Repeated Administration of Diosmin/Hesperidin Combination on the Mechanical and Thermal Hyperalgesia

The DS10/HS100 combination that showed a supra-additive response was chosen to test a repeated treatment by the daily administration of this specific combination for 14 days (Figure 4). Under this condition, the combination reduced the mechanical hyperalgesia shown as a significant enhancement in the withdrawal threshold at days 24 and 28 (Figure 4(c)) and was subjected to a two-way ANOVA. A main effect of dose (*F*_(3,28)_ = 35.374, *P* < 0.001) and time (*F*_(4,28)_ = 2.968, *P* = 0.023) but not of interaction (*F*_(12,28)_ = 1.477, *P* = 0.143) was found. A similar significant response was observed with this treatment and the reference drug (gabapentin at a dosage of 31.6 mg/kg) after repeated administration, both in the withdrawal threshold (Figure 4(c)) and in the withdrawal latency (Figure 4(d)) in comparison to the vehicle group (Figures 4(c) and 4(d)). A two-way ANOVA showed a significant effect of group (*F*_(3,28)_ = 3.212, *P* = 0.038) but not by time (*F*_(4,28)_ = 1.749, *P* = 0.144) and the interaction (*F*_(12,28)_ = 1.638, *P* = 0.091).

### 3.5. Antihyperalgesic Diosmin/Hesperidin Mechanism of Action

Administration of the antagonists* per se* did not modify nociceptive responses in both stimuli, except for haloperidol, which produced a significant increase in the withdrawal threshold at 120 min in comparison to the vehicle group.

**Figure 5:**
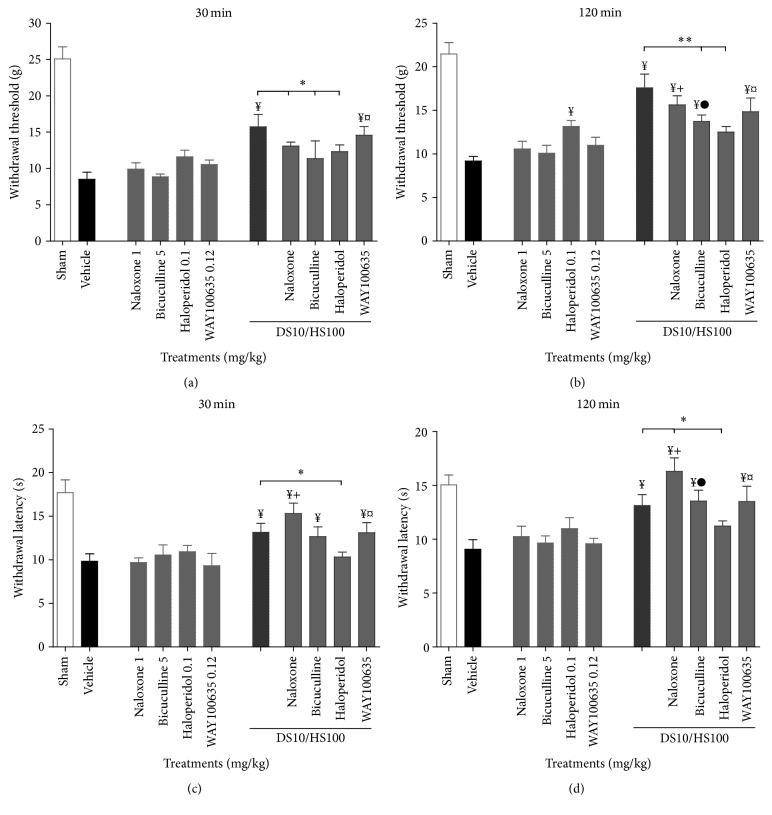
Antihyperalgesic effect obtained from the time course curves of the following compounds alone and in presence of the combination DS10/HS100 mg/kg in the CCI model: naloxone (1 mg/kg, i.p.), bicuculline (5 mg/kg, i.p.), haloperidol (0.1 mg/kg, i.p.), and WAY100635 (0.1 mg/kg, s.c.) on the withdrawal threshold at 30 min (a) and 120 min (b) and withdrawal latency at 30 min (c) and 120 min (d). Each column is the average of withdrawal threshold (g) or withdrawal latency (s) ± SEM in at least six rats. Three-way ANOVA followed by Tukey's test, symbols indicate statistical significance among the DS/HS alone group and the antagonist with DS/HS groups: ^*∗*^ *P* < 0.05, ^*∗∗*^ *P* < 0.01, and ^*¥*^ *P* < 0.001 versus vehicle; ^+^ *P* < 0.001 versus naloxone, ^●^ *P* < 0.001 versus bicuculline, and ^¤^ *P* < 0.001 versus WAY100635.

The significant antihyperalgesic effect produced by the combination of DS10/HS100 in experimental neuropathic rats was reduced in the presence of naloxone (an opioid antagonist, 1 mg/kg, i.p.) for the mechanical stimulus at 30 min (Figure 5(a)). Alternatively, the presence of bicuculline (GABA_A_ antagonist, 5 mg/kg, i.p.) and haloperidol (D_2_ antagonist, 0.1 mg/kg, i.p.) produced inhibition at 120 min after the administration of this combination (Figure 5(b)). A three-way mixed-factor ANOVA revealed significant effect between DS10/HS100 combination alone and in the presence of antagonist (*F*_(1,5)_ = 56.507, *P* < 0.001), antagonists alone and combined with DS10/HS100 treatment (*F*_(5,5)_ = 66.822, *P* < 0.001), and its interaction (*F*_(5,5)_ = 7.328, *P* < 0.001). No significance was obtained with respect to the evaluation time (*F*_(1,5)_ = 0.148, *P* = 0.701) or interactions (Figure 5).

In addition, the antihyperalgesic response of DS10/HS100 was enhanced in the presence of naloxone at 120 min (Figure 5(d)) but inhibited in the presence of haloperidol at 30 min for the thermal stimulus (Figure 5(c)) and without any changes in the presence of bicuculline. On the other hand, the presence of the 5-HT_1A_ antagonist (WAY100635, 0.12 mg/kg, s.c.) did not modify the response in any stimuli with respect to the combination alone. The three-way mixed-factor ANOVA showed a significant effect between combination of DS10/HS100 alone and in the presence of antagonists (*F*_(1,5)_ = 41.154, *P* < 0.001), as well as with antagonists alone and combined with DS10/HS100 (*F*_(5,5)_ = 16.031, *P* < 0.001) and finally with its interaction alone or combined (*F*_(5,5)_ = 5.258, *P* < 0.001) but not by the time (*F*_(1,5)_ = 0.326, *P* = 0.569).

### 3.6. Sedative-Like Effects

Ambulatory activity measured as the total number of squares explored in 3 min in rats receiving DS 10 mg/kg (19 ± 4), HS 100 mg/kg (17 ± 4), the combination DS10/HS100 (19 ± 3), or gabapentin 31.6 mg/kg (27 ± 3) was not modified as compared to the activity in the vehicle group (24 ± 4).

## 4. Discussion

In this study, the potential of hesperidin alone and in combination with diosmin as an option for the treatment of neuropathic pain was investigated. We provided evidence that hesperidin exhibits antihyperalgesic activity improved in the presence of diosmin in the CCI experimental model in rats using two nociceptive tests: paw withdrawal threshold using aesthesiometer and paw withdrawal latency in the Hargreaves procedure. Key results include hesperidin-induced reduction of pain behaviors in both procedures at a dose that did not produce sedative response. Additional studies were conducted to address mechanism of action in the antihyperalgesic effect improved by an interaction between hesperidin and diosmin, both identified at cerebral level.

Due to the similarity of the behavioral responses of the CCI model with the human pathology, it is one of the most frequently used animal models for the study of neuropathic pain and the targets to look for novel treatments [29]. In the present study, CCI induced significant behavioral alterations referred to as mechanical and thermal hyperalgesia. The treatment with either the reference drug gabapentin (31.6 mg/kg, i.p.) or the tested drug hesperidin alone (from 10 to 1000 mg/kg, i.p.) attenuated both mechanical and thermal hyperalgesia in acute treatment. These results are in agreement with recent reports which demonstrate that hesperidin is able to reverse neuropathic pain involving a control over hyperglycemia and hyperlipidemia and downregulation of free radicals generation, as well as a release of proinflammatory cytokines, TNF-*α* and IL-1*β* [17, 30]. In a similar manner, hesperetin (aglycone of hesperidin) produces an antihyperalgesic effect in neuropathic rats involving inhibition of various oxidative markers and proinflammatory mediators secreted at the injury site [24]. Hesperidin also produces diminution in the toxicity of cisplatin in the brain and sciatic nerve by reduction of thiobarbituric acid reactive substances (TBARS) levels, histological changes, electromyographical alteration of the sciatic nerve, and increase in the antioxidant enzyme activities and GSH levels [18]. Other flavonoids have also demonstrated antihyperalgesic efficacy in different experimental models of neuropathic pain such as naringenin [31], luteolin [32], fisetin [33], myricetin [34], myricitrin [35], quercetin [36], and genistein [37, 38]. All these results together reinforce the potential of natural compounds in therapy of this kind of diseases.

Hesperidin is a lipophilic compound that has shown pharmacological central actions as analgesic [26] and anxiolytic-sedative [12] suggesting that it may effectively cross the blood-brain barrier to display central effects. In fact, in the present study, the presence of hesperidin was corroborated in brain homogenates of rats treated by the systemic route of administration, where a portion of the administered hesperidin was metabolized into the flavonoid diosmin. It has already been reported that diosmin can be obtained from the flavonoid hesperidin [39]. In a similar manner, simultaneous determination in human plasma and urine has reported the* in vivo* metabolic reduction of diosmetin to hesperetin [40], the corresponding aglycones of diosmin and hesperidin. So, diosmin can be activated in humans after enzymatic hydrolysis by intestinal microflora and subsequently absorbed into the systemic circulation to produce its activity [41]. This finding suggests that these two flavonoids have a relationship in the organism because of metabolic mechanisms involved in the partial transformation of hesperidin into diosmin and justifying the presence of both in brain samples of rats administered only with hesperidin; therefore, both flavonoids can be responsible for the central effects inhibiting the neuropathic process. Moreover, they may also produce a synergic response mediated by their individual pharmacological mechanisms even at peripheral and central levels.

According to this hypothesis of central activity, in this study we evaluated three combinations of hesperidin plus diosmin taking ineffective dosages that were firstly tested in an individual manner. The three combinations reversed completely the hyperalgesia in the CCI-induced model and overcame antihyperalgesic effects by significant individual doses of both flavonoids, but only a combination of DS10/HS100 mg/kg showed supra-additive effect in mechanical and thermal hyperalgesia. The combination of DS10/HS100 mg/kg was opposite to that applied in the clinic for the treatment of venous insufficiency [7, 19] of which an extensive safety evaluation has found diosmin/hesperidin free from toxicological risk [42]. An advantage in the use of combined therapy is that efficacy can be improved with the simultaneous reduction of adverse effects [43]; this can be possible because of the participation of multiple mechanisms of action avoiding levels of concentration to reach adverse effects. In this respect, no significant effects as sedative-like behavior were observed after administration of the diosmin/hesperidin combination, as it is commonly observed in the therapy of neuropathic pain when treated with gabapentin. Additionally, our data give evidence that a useful combination for venous insufficiency and in hemorrhoidal disease is not necessarily the same amount with possible use to alleviate the neuropathic pain.

Regarding the central neurotransmission in the antihyperalgesic effect of diosmin/hesperidin combination in the CCI model in rats, our results give evidence that this significant antihyperalgesic response is diminished in the presence of the following antagonists: naloxone, bicuculline, and haloperidol, suggesting the participation of opioids, GABA_A_, and D_2_ receptors as partial mechanism in the antinociceptive activity of these combined flavonoids. These results are in agreement with the central activity of these flavonoids as previously described as modulators of GABAergic [12] and opioidergic [14, 15] neurotransmission. However, it is well known that mechanisms of action depend on the experimental condition, such as the model representing a disease.

GABAergic transmission in the spinal cord plays an important role in the nociceptive process in a wide range of pain conditions, such as acute, inflammatory, and even neuropathic pain [44]. The opioid system suppresses nociceptive transmission pre- and postsynaptically at the spinal dorsal horn as does the GABAergic system [45]. Similar actions have been observed with flavonoids such as luteolin and fisetin, which ameliorate mechanical and cold hyperalgesia at least in part by activating GABA_A_ receptors in a flumazenil-insensitive manner and *μ*-opioid receptors in the spinal cord, as well as involving the spinal serotonergic system [32, 33]. However, 5-HT_1A_ receptors were unlikely to contribute to the antihyperalgesic action of hesperidin as observed in this study.

Neuropathic nociception induced by noxious heat and sciatic neurectomy in animals can increase expression of D_2_ dopamine receptor mRNA levels in cg1, suggesting that an expression of D_2_ receptors is partially responsible for the development of autotomy behavior [46]. Several phytochemicals, such as flavanol (−)-epigallocatechin-3-gallate, flavone baicalein, and isothiocyanate sulforaphane, have demonstrated a protective action on dopaminergic neurons and glial cells [47]. Our present results support an involvement of D_2_ dopamine receptors in the antihyperalgesic effect exerted by a DS10/HS100 combination.

## 5. Conclusion

The bioflavonoid hesperidin alleviates the mechanical and thermal hyperalgesia in an experimental model of neuropathic pain. This effect is improved by combination with diosmin. These effects involve central action and the partial participation of dopaminergic, GABAergic, and opioidergic neurotransmission suggesting the potential use of these combined flavonoids in clinical chronic neuropathic pain.

## Abbreviations

CCI: Chronic constriction injury
DS: Diosmin
HS: Hesperidin
UHPLC-MS: Ultra-high performance liquid chromatography coupled masses.
